# Breathable Dry Silver/Silver Chloride Electronic Textile Electrodes for Electrodermal Activity Monitoring

**DOI:** 10.3390/bios8030079

**Published:** 2018-08-24

**Authors:** Peter A. Haddad, Amir Servati, Saeid Soltanian, Frank Ko, Peyman Servati

**Affiliations:** 1 Electrical and Computer Engineering Department, University of British Columbia, Vancouver, BC V6T 1Z4, Canada; saeid@ece.ubc.ca; 2 Materials Engineering Department, University of British Columbia, Vancouver, BC V6T 1Z4, Canada; amirser@mail.ubc.ca (A.S.); frank.ko@ubc.ca (F.K.)

**Keywords:** biomedical, wearable electronics, electrodermal activity, electrodes, flexible electronics, electronic textiles, monitoring

## Abstract

The focus of this study is to design and integrate silver/silver chloride (Ag/AgCl) electronic textile (e-textile) electrodes into different textile substrates to evaluate their ability to monitor electrodermal activity (EDA). Ag/AgCl e-textiles were stitched into woven textiles of cotton, nylon, and polyester to function as EDA monitoring electrodes. EDA stimulus responses detected by dry e-textile electrodes at various locations on the hand were compared to the EDA signals collected by dry solid Ag/AgCl electrodes. 4-h EDA data with e-textile and clinically conventional rigid electrodes were compared in relation to skin surface temperature. The woven cotton textile substrate with e-textile electrodes (0.12 cm^2^ surface area, 0.40 cm distance) was the optimal material to detect the EDA stimulus responses with the highest average Pearson correlation coefficient of 0.913 ± 0.041 when placed on the distal phalanx of the middle finger. In addition, differences with EDA waveforms recorded on various fingers were observed. Trends of long-term measurements showed that skin surface temperature affected EDA signals recorded by non-breathable electrodes more than when e-textile electrodes were used. The effective design criteria outlined for e-textile electrodes can promote the development of comfortable and unobtrusive EDA monitoring systems, which can help improve our knowledge of the human neurological system.

## 1. Introduction

Electronic textile (e-textile) sensors for health monitoring is an area of particular interest for many researchers due to the potential they have for addressing current limitations of existing clinical grade rigid sensors [1,2,3,4]. In particular, flexible and breathable e-textiles can aid in creating a more comfortable and unobtrusive method to monitor biological signals for a variety of applications [5,6,7,8,9,10]. For example, using e-textiles to monitor the neurological health of an individual for a known indicator like electrodermal activity (EDA) [4,11,12,13,14,15,16,17,18,19,20,21,22,23] can potentially help diagnose a medical condition and monitor progress throughout treatment.

EDA is directly related to different states of emotional, cognitive, or physical stress [4]. This response is the result of the sympathetic nervous system (SNS), which is a division of the autonomic nervous system (ANS) [4]. A state of stress causes an increase of sweat on the skin surface and therefore the electrical properties of the skin change, which can be quantitatively monitored [4].

Wearable EDA monitoring devices currently in the market rely on rigid electrodes. Some examples include the Embrace and E4 wristbands by Empatica [24], the Moodmetric ring by Moodmetric [25], and the edaMove by Movisens [26]. There have been significant strides in the development of an e-textile (stainless steel conductor material) EDA monitoring glove, which has identified important electrical parameters and algorithms required to obtain reliable EDA data [11,16,19]. Other research has focused on utilizing silver as the conductive material when monitoring EDA [12,14,15,21,22]. To the best of our knowledge, no previous study exists that compares Ag/AgCl e-textile electrodes against the current clinical grade rigid Ag/AgCl electrodes for EDA monitoring. In addition, previous research utilizing e-textiles for EDA monitoring has also indicated the need for a better understanding of the effect of e-textiles and textiles on EDA monitoring [11,16,22]. A roll-to-roll system for fabrication of Ag/AgCl uniformly coated yarns is developed and used for e-textiles utilized for this work.

Another area of interest in EDA research that still requires a significant amount of study is the concept of asymmetry of EDA on different sides of the human body [27]. This notion of multiple emotional substrates in the brain, which can lead to different innervations on each side of the body, was introduced by Picard et al. in 2016 [27] and has promoted significant dialogue within the research community to ultimately develop a better understanding of EDA as a whole. Early research into understanding the underlying physiology associated with EDA began with animal models [28,29]. In particular, the electrodermal response was shown to be attributed to 60% innervation by the ulnar nerve and 40% innervation by the median nerve in a feline model [28]. In a canine model, it was shown that the thoracic spinal root (T1), which is connected to the SNS, provides input to both the ulnar and median nerves, but the contribution is greater in the former [29]. 

In humans, the little finger is primarily innervated by the ulnar nerve, while the middle and index fingers are mainly innervated by the median nerve, with potential communication between the branches [30]. Also, on the palmar side of the hand, the distal phalanx of all fingers are known to have an increased EDA signal due to an increase in sweat gland density compared to the median and proximal phalanges [31,32]. There is an opportunity to further explore whether there are differences in the EDA responses on the different fingers of the human hand, as there is limited information in previous literature. Therefore, it is believed that through designing, integrating, and testing of Ag/AgCl e-textile electrodes, an understanding of the effect of materials and location of the electrodes on the EDA signal detection can be developed.

## 2. Materials and Methods

### 2.1. Characterization of Textile Substrates

The textile substrates selected to support and integrate the Ag/AgCl e-textile electrodes were 100% cotton, 100% nylon, and 100% polyester, which are all woven fabrics with a plain weave structure (Fabricland, Vancouver, BC, Canada). The threads in the warp and weft direction of each textile substrate were counted with the use of a Leica S9i stereo microscope and the thickness was measured with a micrometer (Mitutoyo Digimatic, MDC-1″ SX, Kawasaki, Japan). Each textile substrate was tested three times for breathability in terms of permeability by following a previous standard protocol by the American Society for Testing and Materials (ASTM) [33] (*n* = 3). Specifically, the water cup testing method was utilized for an eight-hour duration with data collected each hour. A cup (Thwing-Albert Instrument Company, EZ-Cup Vapormeter, West Berlin, NJ, USA) that complies with the ASTM E96 standard was used. The cup, which can hold test samples up to 3 mm thick with a diameter of 63.5 mm, is sealed mechanically with rubber and Teflon. The cup itself has a depth of 19.05 mm and weight of 127.36 g. The chamber used to control airflow was an AirClean System (AC710C, Creedmoor, NC, USA), and a digital hygrometer/psychrometer (TPI 597, Beaverton, OR, USA) monitored temperature and humidity. A weighing balance (Sartorius CPA225D, Bohemia, NY, USA) was used to monitor the change of weight.

The wettability of cotton, nylon, and polyester fabrics, as well as the surface of a solid Ag/AgCl standard electrode, were evaluated by determining the contact angle of a pendant deionized water droplet [34]. A 10 µL water droplet was formed with the use of a 25-gauge needle (305122, BD, Franklin Lakes, NJ, USA) and a 1 mL syringe with tubing. The image to calculate the contact angle of the water droplet was obtained with the use of a high-speed camera (X-PRI, High Speed Imaging, Uxbridge, ON, Canada) and a high intensity illuminator (Fiber-Lite MI-150, Dolan-Jenner Industries, Boxborough, MA, USA). The contact angle of a water droplet was measured on the two sides of the droplet by using Image J software (Version 1.51, National Institutes of Health, Bethesda, MD, USA) and then averaged. Each material was imaged three times (*n* = 3). The material was considered to be hydrophilic if the water droplet contact angle was less than 90° and hydrophobic if the contact angle was greater than 90°.

The bending moment versus curvature curves were plotted for the woven textile substrates and the bending rigidity and hysteresis (recoverability) were determined with a KES-FB2-S bending tester (Kato Tech, Kyoto, Japan) and associated software (KES-FB Measurement Program, Version 8.07, Kyoto, Japan) with a sensitivity of 20 g force·cm [35]. The bending tests were done on 5 cm wide cotton, nylon, and polyester fabrics in the warp and weft directions (*n* = 3). Additionally, the bending displacement velocity was 0.5 cm^−1^/s and the sample in the bending direction was 1.00 cm.

### 2.2. Integration Technique for E-Textile Electrodes with Textile Substrates

**Figure 1:**
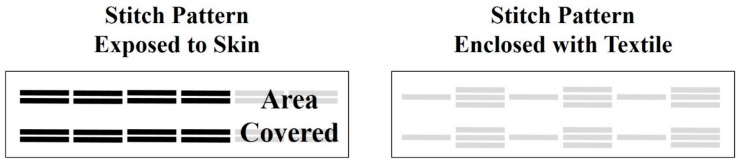
Stitch patterns for e-textile electrodermal activity (EDA) electrodes, where each e-textile electrode exposed to the surface of the skin covers 0.12 cm^2^ of surface area.

The integration of the Ag/AgCl uniformly coated yarns within the textile substrates has gone through various iterations for the final design described in this work. Importantly, the straps with hand-stitched e-textile electrodes were designed to be adjustable for use on the distal phalanx of index, middle, and little fingers. The final design for the EDA e-textile electrode straps, was influenced by the requirement of the electrodes to cover 140 sweat glands to effectively detect EDA stimulus responses, which was outlined in previous work [36]. The final size of the textile substrate was 6 cm long by 1 cm wide, which was specifically intended to be placed on the distal phalanx of the index, middle, and little fingers. The ends of the textile straps have Velcro hooks or pads for easy application or removal of the straps. The e-textile electrodes were hand-stitched with an adapted back stitch, as shown in Figure 1. The two e-textile electrodes exposed to the skin are 2.0 cm long (0.5 cm stitch length) and 0.4 cm apart, with an approximate surface area of 0.12 cm^2^ covered by each electrode. The electrical connector was secured by first stitching it with the e-textile and then coating the conductive yarn on the connector with silver paint (Ted Pella, Redding, CA, USA) and allowing it to dry for 20 min. To encapsulate the yarn and secure the connector, a coating of cyanoacrylate (LePage, Mississauga, ON, Canada) was applied on the silver paint and allowed to dry for 20 min. Six sets of e-textile electrodes for each of the three textile substrates were stitched by hand to assess the success rate of the integration process (*n* = 6). Also, the resistances from the solid electrical connector to the furthest end of a usable e-textile electrode were all measured (*n* = 18).

### 2.3. EDA Monitoring Testing Protocols

The chosen method to detect and record EDA stimulus responses has been previously described in detail in our previous work [36]. Briefly, skin conductance and the EDA response were recorded by utilizing an adapted Stroop color test [37,38]. In addition, a 4-h EDA monitoring experiment was also conducted in which the subject would remain as still as possible during the test. There were no controlled external stimuli provided to the subject and the temperature and humidity of the room were recorded at the beginning and end of the 4-h test with a digital hygrometer/psychrometer.

### 2.4. Systematic Testing of E-Textile and Textile Materials

**Figure 2:**
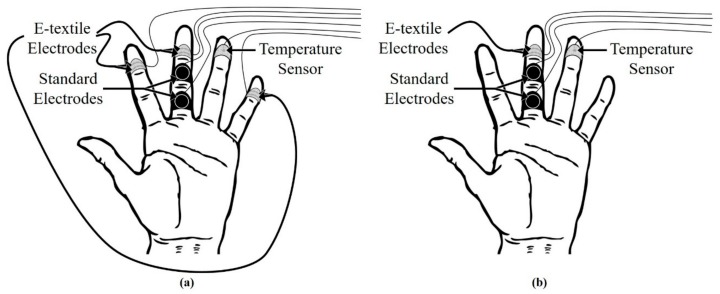
Schematics illustrating the location of the sensors for the (**a**) 15-min tests and (**b**) 4-h test.

The dry Ag/AgCl e-textile EDA electrodes integrated into the cotton, nylon, and polyester substrates were each compared to the standard rigid, non-breathable, and dry Ag/AgCl electrodes simultaneously. Three sets of e-textile electrodes integrated into one type of substrate were attached on the distal phalanx of the index, middle, and little fingers and secured with the use of Velcro. The two conventional solid Ag/AgCl electrodes (Thought Technology, Montreal, QC, Canada) had an approximate surface area of 1.00 cm^2^ each. The conventional rigid Ag/AgCl electrodes were secured on the proximal and medial phalanges of the middle finger with straps and were separated by a distance of 1.50 cm. A compressive force sensor (Pololu, Las Vegas, NV, USA) was used to determine the applied force of the straps or tape on each sensor at the locations shown in Figure 2. The system used to collect the compression force data was an Arduino UNO R3 (Arduino, Turin, Italy) (*n* = 3).

To quantify the sweat gland density of the locations on the palm of the hand of the subject where EDA electrodes were placed, a 10% povidone-iodine topical solution (Betadine Solution, Purdue Pharma, Stamford, CT, USA) with linen paper test was performed [39,40]. The povidone-iodine topical solution was stamped on the location of interest on the palm of the hand of the subject with a 2 by 2 cm piece of linen paper, which was then allowed to dry for approximately 10 min. A clean piece of 2 by 2 cm linen paper was then pressed onto the location of interest for 20 s until small dots appeared on the paper, which indicate the presence of sweat gland ducts. A Leica S9i stereo microscope was used to image the dotted linen paper to count the number of sweat glands per cm^2^ in the area of interest. The sweat gland density of each location where EDA electrodes were placed was tested three times (*n* = 3).

The FlexComp Infiniti (Thought Technology, Montreal, QC, Canada) was used to monitor the EDA signal detected by the conventional and e-textile electrodes. The FlexComp Infiniti system has a sensitivity of 0.01 µS as well as uses DC and constant voltage when measuring skin conductance [22]. The BioGraph Infiniti software was used to sample the data at a rate of 256 Hz for the 15-min tests and at 32 Hz for the 4-h test. For the 15-min tests, the EDA stimulus response was monitored with three e-textile electrodes of a specific textile substrate material (cotton, nylon, or polyester) on the distal phalanx of the index, middle, and little fingers. The EDA stimulus response monitored with the e-textile electrodes was compared to that of the standard electrodes (located on the proximal and medial phalanges of the middle finger). EDA responses were measured three times for each finger location and substrate material, and at no time was a single e-textile strap was used twice in one location (*n* = 3). In order to determine the reliability of the test method, the conventional EDA electrodes were compared against another set of standard EDA electrodes in previous work (*n* = 1) [36]. The average minimum to maximum EDA signal percent change per sweat gland was evaluated to determine if there was a difference in the EDA waveforms recorded on the distal phalanx of the index, middle, and little fingers, regardless of textile substrate used (*n* = 9).

For the 4-h test, the EDA data recorded with a set of e-textile electrodes with a cotton textile substrate on the distal phalanx of the middle finger was compared to the EDA data collected by the standard electrodes on the proximal and medial phalanges of the middle finger (*n* = 1). One subject was used for all tests. The skin surface temperature was also monitored during all experiments with the use of a temperature sensor (Thought Technology, Montreal, QC, Canada).

### 2.5. Statistical Analysis

To compare the EDA stimulus responses recorded during the 15-min tests, a statistical analysis similar to a previous study was done [36]. The EDA responses as a result of the Stroop color test were visually evaluated and sectioned 10 s before and 20 s after the stimulus for the standard electrodes. As the experiments simultaneously recorded the EDA signals with both standard and e-textile electrodes, the EDA stimulus responses detected by the e-textile electrodes were sectioned at the same time points that the EDA stimulus response detected by standard electrodes occurred in order to statistically compare the signals. Importantly, this analysis method included the key aspects of the EDA waveform [4].

The Pearson correlation coefficient [41] was used to compare the EDA response signals detected by e-textile and solid electrodes, a method that has been used before to compare EDA signals [22,36,42]. In order to evaluate the averages and the standard deviations of the Pearson correlation coefficients of different textile substrate materials and locations on the hand that were tested, a one-tailed paired *t*-test was used (*n* = 3). A correlation coefficient of 1 means perfect positive correlation between the signals, while a −1 means perfect negative correlation and the significance level (*p*-value) is less than 0.05 or 0.10. A one-tailed paired *t*-test (*p*-value of less than 0.05) was also conducted to determine significant differences between breathability and wettability results, sweat gland density, and force test results for sensor locations, with standard deviations between tests stated (*n* = 3 for each experimental group). A one-tailed paired *t*-test (*p*-value of less than 0.05) was done on the bending rigidity and hysteresis results of the fabric substrates (*n* = 3).

To further evaluate the effect of location of the e-textile electrodes on the EDA stimulus response waveform, the average percent change from the minimum to maximum conductance across all tests on a specific location was calculated and then divided by the sweat gland density of the location on the finger. A one-tailed paired *t*-test (*p*-value of less than 0.05 or 0.10) was conducted on the average minimum to maximum EDA signal percent change per sweat gland to evaluate if there was a difference in the EDA waveforms recorded on the distal phalanx of the index, middle, and little fingers when corrected for sweat gland density (*n* = 9).

The trends for the 4-h EDA experiment were evaluated based on the signal percent difference of the skin surface temperature, standard electrode baseline EDA signal, and e-textile baseline EDA signal from the beginning of the test and at each hour time point ± 10 min, depending on the time point of the baseline EDA signals. In addition, the Pearson correlation coefficient was calculated with the data for the entire 4-h experiment to compare the EDA signals collected by the standard and e-textile electrodes, the EDA signal of the standard electrodes versus the skin surface temperature, as well as the EDA data recorded by the e-textile electrodes versus skin surface temperature.

## 3. Results

### 3.1. Characterization of Textile Substrates

**Figure 3:**
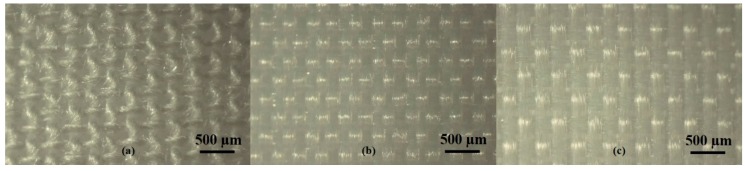
Optical images of (**a**) cotton, (**b**) nylon, and (**c**) polyester fabrics used to estimate the warp and weft thread counts in a 1 × 1 cm area.

The threads in a 1 × 1 cm area in the warp and weft directions of the woven textile substrates of cotton, nylon, and polyester were approximated at 40 × 50, 60 × 40, and 40 × 30, respectively (warp thread count per cm x weft thread count per cm). Optical images obtained of the substrates are shown in Figure 3. The thickness of the cotton, nylon, and polyester substrates are 0.108, 0.097, and 0.065 mm, respectively, and represent important values when calculating the permeability of the textiles. In addition, the chamber temperature of all tests was 22.74 ± 0.10 °C, the average relative humidity was 27.93 ± 0.83% and the air flow was maintained at an average of 0.97 ± 0.00 feet per second (FPS). The permeability of cotton is statistically significantly greater than nylon and polyester (*p*-value < 0.05), and nylon is significantly more permeable than polyester (*p*-value < 0.05). Cotton, nylon, and polyester fabrics have a permeability of 7.02 × 10^−10^ ± 2.69 × 10^−11^ g/Pa·s·m, 5.50 × 10^−10^ ± 1.85 × 10^−11^ g/Pa·s·m, and 4.05 × 10^−10^ ± 1.18 × 10^−11^ g/Pa·s·m, respectively. It is also important to note that standard Ag/AgCl electrodes are solid materials and will therefore have a permeability many orders of magnitude less than textiles.

**Figure 4:**
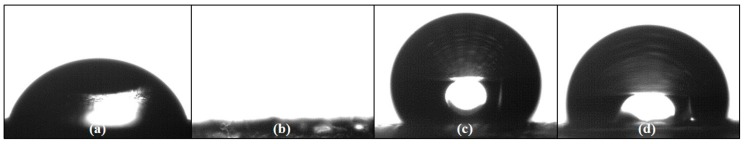
Optical images of water droplet on surfaces of (**a**) solid silver/silver chloride, as well as (**b**) cotton, (**c**) nylon, and (**d**) polyester textiles.

The wettability results of solid Ag/AgCl and cotton, nylon, and polyester textiles are presented in Figure 4. Ag/AgCl and cotton were found to have water droplet contact angles of 60 ± 3° and 0°, respectively, demonstrating hydrophilic properties. Nylon and polyester have water droplet contact angles of 124 ± 1° and 117 ± 2°, confirming their hydrophobic characteristics. All larger average values of the water droplet contact angle for materials are statistically significantly greater than smaller average values of the water droplet contact angles (*p*-value < 0.05).

The average bending rigidity results in the combined warp and weft directions of cotton, nylon, and polyester fabrics were 0.03 × 10^−4^ ± 0.00 × 10^−4^ Nm/m, 0.08 × 10^−4^ ± 0.00 × 10^−4^ Nm/m, and 0.10 × 10^−4^ ± 0.00 × 10^−4^ Nm/m, respectively. The smaller average bending rigidity values were statistically significantly less than the larger values (*p*-value < 0.05). The average bending hysteresis results in the combined warp and weft directions for cotton, nylon, and polyester fabrics were 0.03 × 10^−2^ ± 0.00 × 10^−2^ N/m, 0.05 × 10^−2^ ± 0.00 × 10^−2^ N/m, and 0.01 × 10^−2^ ± 0.00 × 10^−2^ N/m, respectively. The smaller average bending hysteresis values were statistically significantly less than the larger values (*p*-value < 0.05). Lower values for bending rigidity and bending hysteresis indicate that the material being tested is less rigid and has good recoverability, respectively [35]. Therefore, cotton was observed to be the least rigid, while polyester had the best recoverability. Notably, since the solid standard Ag/AgCl electrodes are rigid, they lack the ability to conform to the skin, reducing their potential to be worn comfortably [43].

### 3.2. Evaluation of Integration of E-Textile Electrodes with Textile Substrates

**Figure 5:**
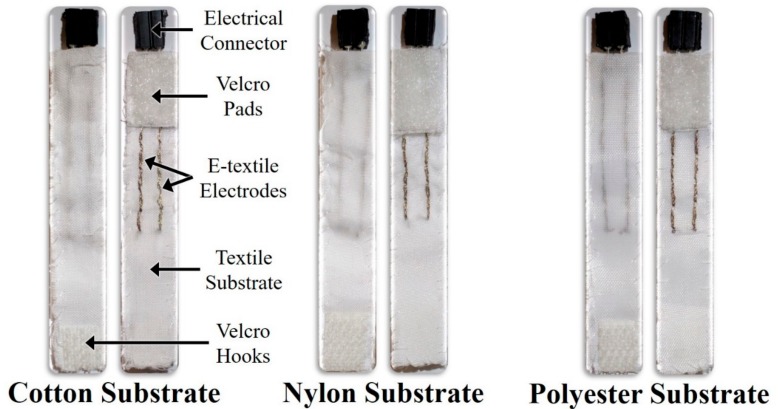
Images of e-textile EDA electrode strap prototypes using cotton, nylon, and polyester textile substrates.

The integration of Ag/AgCl e-textiles with the textile substrates of cotton, nylon, and polyester proved to be a challenge and showed a success rate of approximately 50%. In particular, the connection between the solid electrical components and the conductive yarn was one of the more difficult aspects to accomplish successfully. In addition, since the e-textile EDA electrode straps were developed by hand, the tension of the stitched yarn was difficult to maintain, resulting in a decent amount of variation in the resistance between the electrical connector and the furthermost end of an e-textile electrode, which was on average 80.96 ± 26.53 Ω. The surface area covered under the e-textile electrodes, when added to the area in between e-textile electrodes, was approximately 1.04 cm^2^ for each of the e-textile EDA monitoring straps (see Figure 5 for cotton, nylon, and polyester e-textile EDA electrode strap prototypes). In comparison, the surface area covered under the standard electrodes, when added to the area in between the standard electrodes, was estimated to be 3.96 cm^2^.

### 3.3. Analysis of EDA Stimulus Response Data for E-Textile Electrodes

The results for the average compressive force tests with standard deviations between trials demonstrated that the e-textile EDA electrode straps at the distal phalanx of the index, middle, and little fingers exhibited 0.07 ± 0.01 N, 0.07 ± 0.00 N, and 0.06 ± 0.01 N, respectively. The average force at the distal phalanx of the ring finger with cloth tape for the temperature sensor was 0.07 ± 0.01 N. The straps at the medial phalanx of the middle finger exhibited 0.13 ± 0.02 N of force and at the proximal phalanx of the same finger it was 0.15 ± 0.01 N. Overall, the standard electrode straps were observed to have a statistically significant (*p*-value < 0.05) larger average force on the sensors than the e-textile electrode straps and cloth tape. The sweat gland density of the different locations on the palmar region of the hand in which EDA electrodes were applied represent important values when considering the effects that sweat gland coverage has on the accuracy and precision of EDA stimulus response detection [36]. The average sweat gland density at the distal phalanx of the index, middle, and little fingers are 323 ± 11, 352 ± 3, and 425 ± 9 sweat glands per cm^2^, respectively. The sweat gland density at the medial and proximal phalanges of the middle finger are 233 ± 22 and 255 ± 17 sweat glands per cm^2^, respectively. All larger average values for sweat gland density were statistically significantly greater than the smaller values (*p*-value < 0.05), except when comparing between the results at the medial and proximal phalanges of the middle finger (*p*-value > 0.05). The e-textile EDA electrode straps cover 336 ± 11, 366 ± 3, and 442 ± 9 sweat glands for the distal phalanx of the index, middle, and little fingers, respectively. The standard electrodes cover approximately 966 ± 78 sweat glands for the medial and proximal phalanges of the middle finger.

**Figure 6:**
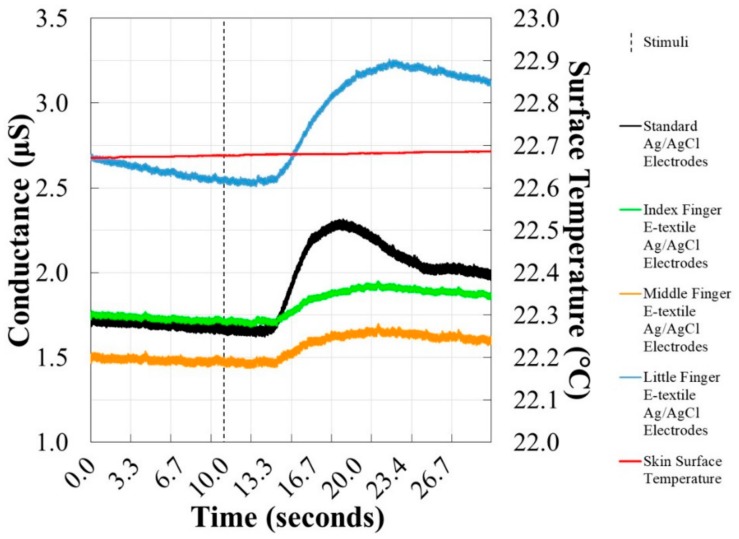
Example EDA stimulus response shown by conductance (primary *y*-axis) for dry e-textile electrodes (0.12 cm^2^ surface area, 0.40 cm distance) at the distal phalanx of the index, middle, and little fingers compared to dry commercial electrodes (1.00 cm^2^ surface area, 1.50 cm distance) on the proximal and medial phalanges of the middle finger. Skin surface temperature is also shown (secondary *y*-axis).

**Figure 7:**
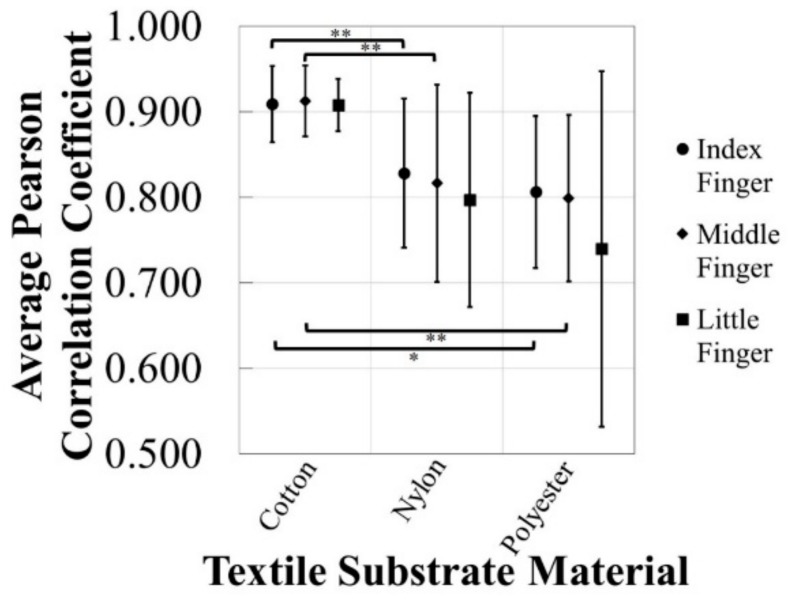
The average Pearson correlation coefficients for the comparison of the EDA stimulus responses of the rigid electrodes (1.50 cm distance, 1.00 cm^2^ surface area) to the signals of the e-textile electrodes of 0.12 cm^2^ surface area with distances of 0.40 cm integrated into cotton, nylon, and polyester textile substrates on the distal phalanx of the index, middle, and little finger on the palm of the hand. Standard deviations are shown and one-tailed *t*-test results with *p*-values < 0.05 (*) and *p*-value < 0.10 (**) are indicated. For each value *n* = 3.

The 15-min EDA stimulus response detection tests were done at room temperature (approximately 25 °C) and the average skin surface temperature recorded with a standard deviation between the EDA monitoring trials was 22.76 ± 0.70 °C. An example of an EDA stimulus response detected with e-textile electrodes on a cotton substrate at different locations on the palm of the hand is presented in Figure 6. The EDA stimulus responses detected by e-textile electrodes were compared to those recorded by standard solid electrodes in order to observe differences between the materials used, as well as the locations of the e-textile electrodes on the palm of the hand. The Pearson correlation coefficients for the comparison of EDA stimulus response signals are shown in Figure 7. In previous work, two sets of standard electrodes were tested to confirm the reliability of the testing method, which resulted in a Pearson correlation of 0.998 [36].

The cotton textile substrate on the distal phalanx of the middle finger was found to have the highest average Pearson correlation coefficient (0.913 ± 0.041), when evaluating all of the e-textile EDA electrode straps and locations. The average correlation coefficient when utilizing the e-textile EDA electrodes integrated into the cotton textile substrate on the distal phalanx of the index finger (0.909 ± 0.044) was statistically significantly greater than the correlation coefficient of the e-textile EDA electrodes integrated into the polyester textile substrate at the same location (0.807 ± 0.089), with a *p*-value of 0.03. Also, the correlation coefficient from e-textile EDA electrodes integrated into the cotton textile substrate on the distal phalanx on the index finger (0.909 ± 0.044) was statistically significantly greater than the correlation coefficient of the e-textile EDA electrodes integrated into the nylon textile substrate at the same location (0.828 ± 0.087) with a *p*-value of 0.08. The e-textile EDA electrodes incorporated with the cotton textile substrate on the distal phalanx of the middle finger had a Pearson correlation coefficient (0.913 ± 0.041) statistically significantly greater than both the e-textile EDA electrodes stitched into the nylon (0.817 ± 0.115) and polyester (0.799 ± 0.097) textile substrates on the same finger, with *p*-values of 0.08 and 0.06, respectively. With this analysis, there were no statistically significant differences observed with the different textile substrate materials on the little finger or between different finger locations of e-textile EDA electrodes. The most notable result is that the cotton textile substrate with integrated Ag/AgCl e-textile EDA electrodes is the most accurate and precise e-textile strap tested when comparing the EDA stimulus response signal to that of the standard solid Ag/AgCl electrodes. In addition, with the cotton textile substrate, there appears to be a significant improvement in the repeatability between trials with Ag/AgCl e-textile electrodes, as the standard deviations on each finger are less than when nylon and polyester textile substrates are used.

**Figure 8:**
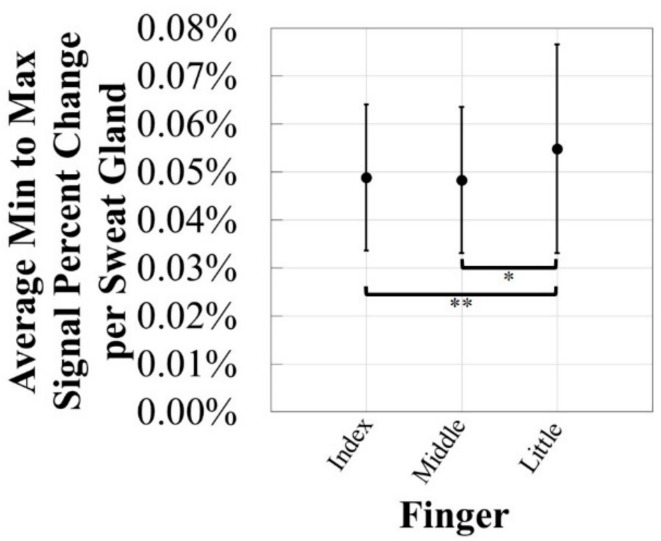
The average minimum to maximum EDA signal percent change per sweat gland for comparison of EDA waveforms from the distal phalanx of the index, middle, and little fingers on the palm. Standard deviations are shown and one-tailed *t*-test results with *p*-values < 0.05 (*) and *p*-value < 0.10 (**) are indicated. For each value *n* = 9.

To further analyze whether the location of the e-textile EDA electrodes has an effect on the EDA stimulus response, the average minimum to maximum EDA signal change was evaluated when the electrodes were on the distal phalanx of the index, middle, and little fingers, regardless of which textile substrate was used. The percent change was then corrected by the sweat gland density of the location of the e-textile EDA electrodes in order to observe if there was a potential for other factors to influence a difference in EDA stimulus response waveforms. As shown in Figure 8, the average EDA signal percent change per sweat gland on the distal phalanx of the little finger (0.055 ± 0.022% per sweat gland) was statistically significantly greater than on the distal phalanx middle finger (0.048 ± 0.015% per sweat gland), with a *p*-value of 0.03. In addition, the average EDA signal percent change per sweat gland on the distal phalanx of the little finger (0.055 ± 0.022% per sweat gland) was also statistically significantly greater than that on the distal phalanx index finger (0.049 ± 0.015% per sweat gland), with a *p*-value of 0.06. These results indicate that there may be underlying factors that affect the EDA waveform other than the sweat gland density of the specific location being monitored. Longer-term experiments were then conducted on the e-textile EDA electrodes integrated into the cotton textile substrate, as these straps were shown to detect the most accurate and precise EDA stimulus response. The location for further EDA testing of the e-textile EDA electrodes was chosen to be the distal phalanx of the middle finger, as the standard EDA electrodes were also located on the middle finger. Choosing this experimental set-up eliminated the potential effects that the location of electrodes could have on the EDA signal.

### 3.4. Analysis of 4-h EDA Data for E-Textile Electrodes

**Figure 9:**
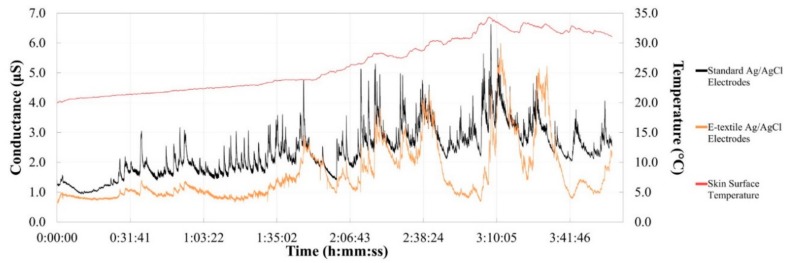
4-h EDA data shown by conductance (primary *y*-axis) for dry e-textile electrodes (0.12 cm^2^ surface area, 0.40 cm distance) on the distal phalanx of the middle finger compared to dry commercial electrodes (1.00 cm^2^ surface area, 1.50 cm distance) on the proximal and medial phalanges of the middle finger. Skin surface temperature is also shown (secondary *y*-axis) with time in seconds (*x*-axis).

**Figure 10:**
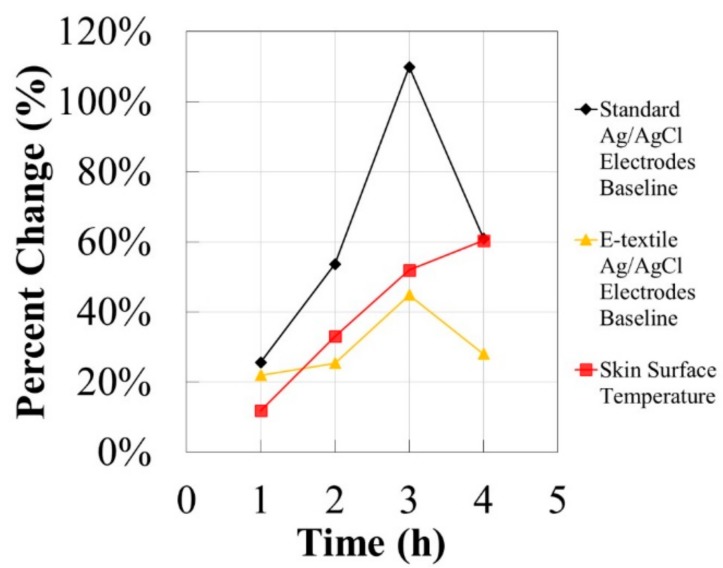
The signal percent difference of the skin surface temperature, standard electrode baseline EDA signal, and e-textile baseline EDA signal from the beginning of the test and at each hour time point ± 10 min.

The EDA data recorded over 4 h with dry Ag/AgCl e-textile EDA electrodes integrated into a cotton textile substrate was compared to the EDA signal collected by dry standard solid Ag/AgCl electrodes. In addition, the EDA signals of both sets of electrodes were compared against the changing skin surface temperature. Figure 9 shows the EDA and temperature signals collected during the 4-h test. Importantly, the change in all signals was evaluated across the 4-h test to determine if there were observable trends with the data collected. The room, which had an open window, had a temperature and relative humidity at the beginning of the test to the end of approximately 23 °C and 26% to 30 °C and 17%, respectively. The signal percent difference of the skin surface temperature, standard electrode baseline EDA signal and e-textile baseline EDA signal from the beginning of the test and at each hour time point ± 10 min is presented in Figure 10. The trends observed indicate that the baseline EDA signal of the standard non-breathable and non-flexible electrodes is affected by the change in temperature more than the baseline EDA signal of the e-textile electrodes. Specifically, at the 4-h time point, it is observed that the skin surface temperature has increased by 60%, the baseline EDA of the standard electrodes has increased by 61%, while the baseline EDA e-textile electrodes increased by 28%. Similar results are also observed at other time points. At the 4 h time point shown in Figure 10, a decrease was observed in both EDA signals, but there was an increase in the skin surface temperature. These contrasting changes could be attributed to a physiological change in the EDA baseline signal that is not related to the skin surface temperature, since EDA is mainly a behavioral response.

The Pearson correlation coefficients of the complete 4-h test were calculated to compare the EDA signals collected by the standard and e-textile electrodes, the EDA signal of the standard electrodes versus the skin surface temperature, as well as the EDA data recorded by the e-textile electrodes versus skin surface temperature. The EDA signals collected by the standard and e-textile electrodes showed a relatively high correlation coefficient of 0.746, considering the length of the test. The EDA data recorded by the standard electrodes also showed a relatively high correlation coefficient of 0.746 when compared to the skin surface temperature signal. A lower correlation coefficient of 0.569 was obtained when comparing the EDA signal measured by the e-textile electrodes and the skin surface temperature data. These results demonstrate a trend that the EDA signal recorded by standard electrodes are more correlated to the skin surface temperature signal than that of e-textile electrodes. Also, future research can explore these trends and should utilize a room with controlled temperature and humidity in order to allow for the repeatability of the testing conditions.

## 4. Discussion

The design and development of functional Ag/AgCl e-textile EDA electrodes was detailed, and the effect of textile substrate materials on monitoring EDA was evaluated. After establishing a practical design for e-textile EDA electrode straps, it was observed that the optimal tested woven textile substrate material to accurately and precisely detect an EDA stimulus response, comparable to signals recorded by standard solid EDA electrodes, is cotton. It was also shown that the EDA waveforms recorded on the distal phalanx of the little finger are different when compared to those recorded on the distal phalanx of the index and middle finger when correcting for sweat gland density at each location. This provides additional discussion points to further the conversation regarding EDA asymmetry in the literature [27], as well as the effect of nerve innervations on EDA [28,29]. In addition, for a 4-h EDA monitoring experiment, a trend indicated that the EDA signal of standard electrodes seem to be more affected by changes in skin surface temperature than the EDA data collected by e-textile electrodes. Each of these conclusions can impact the development approach of future e-textile systems for monitoring EDA. This work outlines important e-textile design criteria for accurate and precise EDA recording, which has the potential to lead to the creation of fully wearable and comfortable long-term e-textile EDA monitoring devices.

The conductive materials for e-textiles that have been used to detect EDA signals include steel [11,16,19,44,45] and silver [12,14,15,21,22], but no study exists in the EDA monitoring research that compares Ag/AgCl coated e-textiles with the standard Ag/AgCl electrodes used clinically. The clinical standard electrodes are made of Ag/AgCl specifically because they have a low electrode-skin impedance, low noise, and minimal motion artifact when compared to polarizable electrodes such as those made of stainless steel [46]. It is, however, important to note that Ag/AgCl is known to be vulnerable to deterioration if not carefully maintained [4,23,46]. Ag/AgCl is importantly a non-polarizing material, therefore when monitoring EDA, which is dependent on an electrolyte (sweat), using an electrode material that allows for current to cross the electrode-electrolyte interface can allow for reliable long-term EDA signal detection, especially when DC is utilized [4,23,46]. One of the major reasons for utilizing Ag/AgCl e-textile electrodes in this work is specifically because a comparable material to the standard solid Ag/AgCl electrodes was required in order to more reasonably conclude that the results observed were not due to the use of different conductive materials, but because of the different properties of e-textiles, textiles, and solid materials.

Effective EDA stimulus response detection was obtained with e-textile electrodes and, as previously described, the cotton textile substrate was identified as the best material to use for the e-textile integrated EDA electrodes when compared to nylon and polyester textile substrates. Particular differences between the cotton, nylon, and polyester textiles, which could be attributed to the differences observed when monitoring EDA stimulus responses, included wettability and breathability. Also, cotton was shown to be the least rigid of the fabrics, potentially allowing this textile substrate to better conform to the surface of the skin. A major difference between the textile substrates is that cotton is hydrophilic and absorbs water, while nylon and polyester are specifically hydrophobic. Importantly, solid Ag/AgCl material was shown to be hydrophilic, but without fully absorbing water. The skin-electrode interface is a critical component in accurately detecting an EDA stimulus response [4,23]. In particular, if there is a disturbance of the electrolytes near the solid-liquid electrode interface, artifacts in the signal can occur [4,23]. The cotton textile substrate is able to maintain a relatively more stable solid-liquid interface, due to being hydrophilic and absorbing sweat, when compared to nylon and polyester. The hydrophilic property and improved bendability of the cotton textile substrate is believed to enable better detection of the EDA stimulus response than when nylon or polyester is used. Also, the EDA signal collected by Ag/AgCl e-textile electrodes integrated into the cotton textile substrate is comparable to the EDA data recorded by solid Ag/AgCl electrodes, which were shown to be hydrophilic as well. Importantly, one of the main advantages of the Ag/AgCl e-textile electrode is the ability to bend and conform to surface of the skin, which increases comfort but also can improve functionality of the sensor for long-term monitoring. Solid conventional electrodes lack this ability to fully conform to the skin and are therefore much more limited in terms of the locations in which they can be placed on the human body.

The slight but statistically significant difference in the EDA waveform monitored on the distal phalanx of the little finger, when compared to the distal phalanges of the middle and index fingers, was observed irrespective of the different sweat gland density at each location. Previous literature showed the amplitude of EDA stimulus responses measured between the distal phalanx of the thumb and the medial phalanx of the little finger to be approximately 1.53 times larger than the EDA responses measured between the distal phalanx of the index and middle fingers [47]. Ranogajec and Geršak in 2014 compared different locations on the hand for EDA monitoring and utilized solid Ag/AgCl electrodes with isotonic gels to highlight the differences in amplitude of skin conductance at these different locations [47]. Importantly, the work presented identifies a previously unknown potential difference between EDA stimulus response waveforms recorded exclusively on the little finger when compared to the middle and index fingers. This difference was observed when correcting for the varying sweat gland densities of the finger locations tested, suggesting other physiological factors could be influencing the EDA waveforms. Specifically, it is possible that the little finger being innervated primarily by the ulnar nerve and the middle and index fingers being innervated mainly by the median nerve could influence the EDA response at the different locations on the hand. The morphology of the EDA waveforms detected by e-textile and standard electrodes also appear to be slightly different, potentially due to the material properties of each type of electrode. Specifically, the e-textile electrodes can absorb the sweat more readily, which can potentially cause a longer recovery time for the EDA response. Further testing on multiple subjects to explore the difference of EDA signals on the different fingers of the hand is justified and required to make more inferences on the underlying physiology associated with EDA, as there are known variations between individuals in regards to communication branches in the nerves located in the hand [30]. Also, increasing the sample sizes of our experiments with a multiple subject approach is justifiable and would promote further understanding of the statistically significant results that we have obtained in this work.

There has been a significant amount of previous research on the effect of ambient and skin surface temperature when monitoring EDA [48,49,50,51], however none of these studies tested the use of e-textile EDA electrodes. The 4-h experiment assessing e-textile electrodes with the cotton textile substrate developed in our work was conducted in order to identify trends with the EDA data over an extended time. In particular, the EDA signals of the Ag/AgCl e-textile electrodes and the standard solid Ag/AgCl electrodes were evaluated against the skin surface temperature. The trend observed was that the EDA baseline signal from the standard solid electrodes was more affected by the increase in skin surface temperature than the baseline signal from the breathable and flexible e-textile electrodes. In addition, the EDA signal of the standard electrodes was more closely correlated to the skin surface temperature data than that of the e-textile electrodes. It is believed that the breathability of the cotton substrate allowed the sweat on the surface of the skin to evaporate more freely than that of non-breathable standard electrodes, reducing the drift in the EDA baseline signal, which is known to occur with increasing moisture on the skin under the EDA electrodes, most commonly caused by additional electrolyte cream [4,23]. The drift in baseline EDA was also seen in this study when utilizing dry solid electrodes, with the electrolyte solution being the sweat generated by the subject. Also, it is important to note that by not using an electrolyte cream, the recording conditions could become more uncontrollable [4,23]. However, by using a textile cotton substrate that is permeable to water and sweat, there is a potential to be able to record a more biologically accurate EDA signal, as the surface of the skin would be able to function more naturally than if covered with non-permeable materials, electrolyte gels, and skin irritating adhesives. 

Similar to previous work [36], a one-subject approach was used for evaluating the e-textile electrodes and textile substrates for EDA monitoring in order to reduce the inter-subject variation associated with a multi-subject experimental design [52]. The goal was to focus on the development, design, and functionality of e-textile electrodes, and the current results of our research justify additional studies on multiple subjects, especially when developing a further understanding of the physiological aspects associated with EDA signals and on the asymmetrical responses across the body. Further research that is focused on the electrical specifications for EDA, similar to work outlined by Greco et al. in 2016 [11], is necessary to advance alternative monitoring methods for EDA other than the most common two electrode system with DC and constant voltage [4,23]. Our study used the most common method for monitoring EDA with a clinical grade Thought Technology system, as the focus of our work was on comparing our developed e-textile EDA electrode strap prototypes to current clinical standard electrodes.

## 5. Conclusions

The textile substrates used to prototype Ag/AgCl e-textile EDA electrode straps were characterized to develop a practical final design for the prototypes. The dry e-textile EDA electrodes were stitched into woven cotton, nylon, and polyester textile substrates, where hydrophilic cotton was demonstrated to be the most accurate and precise substrate used to detect EDA stimulus responses when compared to EDA signals recorded simultaneously by dry standard solid Ag/AgCl electrodes. The EDA waveform recorded by e-textile EDA electrodes on the little finger was shown to be different from those detected on the index and middle fingers, potentially a result of different nerve innervations on the human hand. 4-h EDA monitoring tests were also conducted and showed a trend which indicated that the EDA signal recorded by solid standard Ag/AgCl electrodes EDA is more affected by changes in skin surface temperature than Ag/AgCl e-textile EDA electrodes. The development of breathable and flexible Ag/AgCl e-textile electrode straps that can monitor EDA comparably to standard solid non-breathable Ag/AgCl electrodes is an important technological step. The identified effective design criteria for e-textile EDA electrode straps detailed in this work can help advance the comfort and wearability of long-term EDA monitoring systems, to ultimately advance our knowledge of the human neurological system. By enabling effective long-term EDA data collection, wearable devices can potentially identify EDA as an important clinical biomarker, which can help improve neurological disorder treatment monitoring. This further understanding of EDA could have a direct positive impact on individuals with autism [53], Parkinson’s disease [54], and epilepsy [17].
